# Periprocedural body temperature and clinical outcomes after successful reperfusion in large vessel occlusion stroke

**DOI:** 10.3389/fneur.2026.1910898

**Published:** 2026-10-09

**Authors:** Hong-Ting Yan, Qi Wang, Lu Zhang, Gao-Peng Xing, Jia-Qi Wang, Yue Wang, Hui-Sheng Chen

**Affiliations:** 1 Department of Graduate School, Dalian Medical University, Dalian, China; 2 Department of Neurology, General Hospital of Northern Theater Command, Shenyang, China

**Keywords:** acute ischemic stroke, body temperature, endovascular treatment, functional outcome, stroke severity

## Abstract

**Background:**

Functional recovery remains heterogeneous among patients with large-vessel occlusion stroke, even after successful reperfusion. Body temperature may constitute a potentially modifiable physiological factor associated with functional outcome. In particular, whether clinical factors, including baseline stroke severity, modify this temperature-outcome association remains uncertain.

**Methods:**

We retrospectively analyzed 219 patients with large-vessel occlusion acute ischemic stroke (LVO-AIS) who underwent EVT and achieved successful reperfusion, defined as a modified Thrombolysis in Cerebral Infarction score of 2b-3. Pre and post-EVT body temperatures were recorded. The primary outcome was favorable functional outcome, defined as a modified Rankin Scale (mRS) score of 0-2 at 90 days. Logistic regression analyses and exploratory subgroup analyses were performed to evaluate the association between body temperature and clinical outcomes.

**Results:**

Among the 219 included patients, 104 (47.5%) achieved favorable functional outcome at 90 days. Pre-EVT temperature was not significantly associated with functional outcome. In unadjusted analysis, each 1 °C increase in post-EVT temperature was associated with lower odds of favorable functional outcome (OR, 0.40; 95% CI, 0.20-0.79; *P* = 0.01) and higher odds of mortality (OR, 2.02; 95% CI, 1.11-3.66; *P* = 0.02). However, these associations were attenuated after multivariable adjustment (adjusted OR, 0.47; 95% CI, 0.19-1.15; *P* = 0.10; adjusted OR, 1.22; 95% CI, 0.49-3.02; *P* = 0.67). In exploratory subgroup analyses, a potential association between higher post-EVT temperature and lower odds of favorable outcome was observed in patients with baseline NIHSS ≤ 15 (adjusted OR, 0.29; 95% CI, 0.08-0.98; *P* = 0.047), although the interaction was not statistically significant (*P* for interaction = 0.64).

**Conclusion:**

In this exploratory study, body temperature was not independently associated with functional outcome in the overall cohort. Although a potential association between higher post-EVT temperature and unfavorable outcome was observed in patients with baseline NIHSS ≤ 15, the interaction was not significant. This finding is hypothesis-generating and warrants prospective validation with pre-specified subgroup analyses.

## 1 Introduction

Acute ischemic stroke (AIS) remains a leading cause of death and long-term disability worldwide (1, 2). Endovascular treatment (EVT) has become the standard treatment for large-vessel occlusion (LVO) stroke (3–5). However, successful reperfusion does not ensure favorable functional recovery (6, 7). Reperfusion injury is increasingly recognized as an important contributor to poor outcomes after EVT (8–10).

Body temperature is a modifiable physiological factor that may influence ischemia-reperfusion injury (11). Experimental studies have shown that hyperthermia exacerbates metabolic stress and inflammation, whereas hypothermia exerts neuroprotective effects (12–15). However, despite promising preclinical evidence, therapeutic hypothermia has not consistently improved outcomes in clinical stroke studies (16–18). One possible explanation is that the biological impact of temperature may not be uniform across all patients and may vary according to stroke severity.

Despite previous studies reporting associations between body temperature and outcomes, whether body temperature independently influences outcomes after successful reperfusion remains unclear. In many prior cohorts, patients with failed recanalization were also included, making it difficult to distinguish temperature-related reperfusion injury from the overwhelming effect of persistent ischemia. Moreover, it remains unknown whether the prognostic relevance of body temperature differs according to stroke severity.

This exploratory study investigated the association between peri-procedural body temperature and functional outcomes in a homogeneous cohort of successfully reperfused LVO-AIS patients, with particular attention to potential heterogeneity according to baseline stroke severity.

## 2 Methods

### 2.1 Study design and population

This was a retrospective analysis based on DETECT-China (NCT04752735, Registration Date: February 10, 2021), which evaluates the efficacy and safety of EVT in patients with acute large vessel occlusion across 24 comprehensive stroke centers in China. For the present analysis, periprocedural temperature data were available only for patients enrolled at the General Hospital of Northern Theater Command, as the corresponding data from other participating centers were not accessible to the study team. The present study was therefore conducted as a single-center analysis (Figure 1).

**Figure 1:**
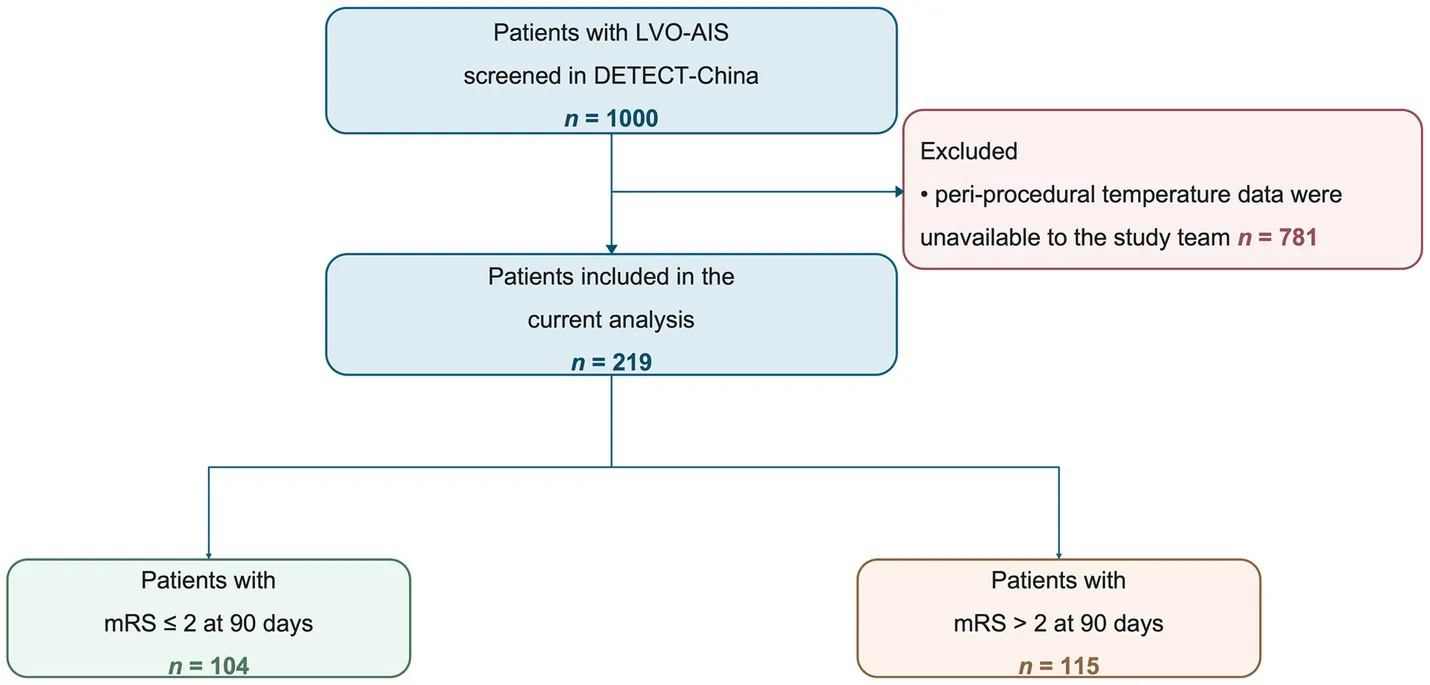
Flowchart of patient selection. LVO-AIS, large-vessel occlusion acute ischemic stroke; mRS, modified Rankin Scale.

Patients were included if they met the following criteria: (1) age ≥ 18 years; (2) underwent EVT within 24 hours of symptom onset; (3) achieved successful reperfusion, defined as modified Thrombolysis in Cerebral Infarction (mTICI) score 2b-3; (4) pre-stroke modified Rankin Scale (mRS) score ≤ 1. Exclusion criteria were: (1) pre-stroke disability (mRS ≥ 2); (2) unsuccessful recanalization (mTICI score 0-2a); (3) missing 90-day mRS follow-up or missing temperature measurement data. The study was conducted in accordance with the Declaration of Helsinki and approved by the regional institutional ethics committee. Due to the retrospective nature of the study, informed consent was waived.

### 2.2 Data collection and assessment

Data collected from medical records included demographic characteristics, vascular risk factors, and laboratory parameters obtained on admission before post-EVT temperature monitoring, procedural details recorded during EVT, and medical complications and antibiotic administration documented during hospitalization. Stroke onset time was defined as the time the patient was last known to be well. Neurological deficit at admission was assessed using the National Institutes of Health Stroke Scale (NIHSS). The extent of baseline ischemia was evaluated on initial non-contrast CT using the Alberta Stroke Program Early CT Score (ASPECTS). Stroke etiology was classified according to the Trial of Org 10172 in Acute Stroke Treatment (TOAST) criteria. Functional outcome at 90 days was assessed using the modified Rankin Scale (mRS) through in-person visits or structured telephone interviews conducted by certified neurologists. All clinical and imaging evaluations were performed by trained assessors who were blinded to the study hypotheses and independent of the study team.

### 2.3 Temperature measurement and outcomes

Body temperature was routinely measured by trained nursing staff using a thermometer (CEM, China). All measurements were performed at standardized time points in accordance with the manufacturer’s instructions and institutional nursing procedures. Temperature measurements were obtained on admission and every 4 hours after EVT. The highest recorded temperature within 24 hours after EVT was used as the post-EVT body temperature. Temperature data were retrospectively extracted from electronic medical records. Antipyretic treatment was administered according to routine institutional practice when clinically indicated.

The primary outcome was favorable functional outcome, defined as mRS 0-2 at 90 days post-stroke. The secondary outcomes included any intracranial hemorrhage (ICH), all-cause mortality, and the ordinal shift of mRS scores at 90 days.

### 2.4 Statistical analysis

Categorical variables were summarized as counts and percentages, while continuous variables were expressed as medians with interquartile ranges (IQRs) because they were not normally distributed. Comparisons between groups were performed using the chi-square test for categorical variables and the Mann-Whitney U test for continuous variables. Complete-case analyses were performed, and no imputation for missing data was applied.

The association between body temperature and 90-day functional outcome (mRS 0-2 versus > 2) was evaluated using binary logistic regression, with temperature treated as a continuous variable. Results were reported as odds ratios (ORs) and 95% confidence intervals (CIs) per 1°C increase. Covariates included in the multivariable model were selected based on clinical relevance. The adjusted variables included age, sex, smoking, hypertension, diabetes, baseline NIHSS score, ASPECTS, anterior circulation occlusion, TOAST classification, and white blood cell count at admission.

Exploratory subgroup analyses included age, sex, baseline NIHSS score, and occlusion location. Age and baseline NIHSS score were divided at the median values of the overall study population. Interactions between variables were evaluated on a multiplicative scale, and *P* values for interaction were calculated using the multiplicative model.

All statistical tests were two-tailed, and *P* < 0.05 was considered statistically significant. Analyses were conducted using SPSS version 25.0 (IBM Corporation, Armonk, NY) and R software version 4.1.2 (R Foundation for Statistical Computing, Vienna, Austria).

## 3 Results

A total of 219 patients with large-vessel occlusion stroke who underwent EVT were included in the analysis (Table 1). The median age was 65 years (IQR 56-72), and 164 patients (74.9%) were male. The median baseline NIHSS score was 15 (IQR 12-18). The majority of patients had anterior circulation occlusions (73.1%). At 90 days, 104 patients (47.5%) achieved favorable outcomes (mRS 0-2). The median pre-EVT temperature was 36.5 °C (IQR 36.3-36.6 °C), and the median post-EVT temperature was 36.6 °C (IQR 36.5-36.8 °C).

**Table 1:** Baseline, procedural, and outcome parameters.

	All patients (*n* = 219)	mRS at 90 days		*P* value
		mRS ≤ 2 (n *=* 104)	mRS > 2 (*n* = 115)	
Demographics and clinical data				
Age, y	65 (56-72)	63.5 (56-71)	66 (55-74)	0.22
Sex (male)	164/219 (74.9%)	73/104 (70.2%)	91/115 (79.1%)	0.13
Risk factors				
Smoking	123/219 (56.2%)	59/104 (56.7%)	64/115 (55.7%)	0.87
Hypertension	132/219 (60.3%)	60/104 (57.7%)	72/115 (62.6%)	0.46
Diabetes mellitus	53/219 (24.2%)	19/104 (18.3%)	34/115 (29.6%)	0.05
Atrial fibrillation	53/219 (24.2%)	29/104 (27.9%)	24/115 (20.9%)	0.23
Prior stroke	64/219 (29.2%)	29/104 (27.9%)	35/115 (30.4%)	0.68
Index stroke data				
Systolic blood pressure, mmHg	148 (134-166)	148.5 (133.5-164.25)	149 (134-169.75)	0.91
Heart rate, bpm	78 (66-88)	73.5 (65-89.5)	78 (68-88)	0.25
Baseline NIHSS	15 (12-18)	13 (10-16)	17 (13-23)	<0.01*
ASPECTS	8 (7-10)	9 (8-10)	8 (7-10)	0.06
Anterior circulation occlusion	160/219 (73.1%)	83/104 (79.8%)	77/115 (67.0%)	0.03*
TOAST				
Large-artery atherosclerosis	138/216 (63.9%)	63/104 (60.6%)	75/112 (67%)	0.27
Cardioembolism	58/216 (26.9%)	33/104 (31.7%)	25/112 (22.3%)	
Undetermined	20/216 (9.3%)	8/104 (7.7%)	12/112 (10.7%)	
Infarct location				
Cortical	26/219 (11.9%)	15/104 (14.4%)	11/115 (9.6%)	0.14
Subcortical	38/219 (17.4%)	21/104 (20.2%)	17/115 (14.8%)	
Cortical plus subcortical	96/219 (43.8%)	47/104 (45.2%)	49/115 (42.6%)	
brainstem/cerebellum	59/219 (26.9%)	21/104 (20.2%)	38/115 (33%)	
Artery occlusion site				
MCA	95/219 (43.4%)	55/104 (52.9%)	40/115 (34.8%)	0.04*
ACA	39/219 (17.8%)	18/104 (17.3%)	21/115 (18.3%)	
BA	51/219 (23.3%)	20/104 (19.2%)	31/115 (27%)	
VA	7/219 (3.2%)	1/104 (1.0%)	6/115 (5.2%)	
Multiple vessel occlusion	27/219 (12.3%)	10/104 (9.6%)	17/115 (14.8%)	
IV thrombolysis	16/219 (7.3%)	11/104 (10.6%)	5/115 (4.3%)	0.08
Procedural measures				
OPT, min	407 (274-548)	388.5 (260.75-562)	425 (287-530)	0.65
DPT, min	44 (34-70)	44 (33.75-78.5)	45 (35-64.25)	0.9
ORT, min	487 (340-643)	478 (326.25-651.25)	495 (354-615)	0.45
Number of passes	1 (1-2)	1 (1-2)	1 (1-2)	0.33
Residual stenosis	99/219 (45.2%)	47/104 (45.2%)	52/115 (45.2%)	0.99
Body temperature, °C				
pre-EVT	36.5 (36.3-36.6)	36.5 (36.3-36.6)	36.5 (36.2-36.6)	0.3
post-EVT	36.6 (36.5-36.8)	36.5 (36.4-36.8)	36.6 (36.5-36.8)	0.07
Medical complications				
Pneumonia	128/219 (58.4%)	44/104 (42.3%)	84/115 (73.0%)	<0.01*
Urinary tract infection	24/219 (11.0%)	12/104 (11.5%)	12/115 (10.4%)	0.79
Sepsis	3/219 (1.4%)	1/104 (1%)	2/115 (1.7%)	0.62
Administration of antibiotics	134/219 (61.2%)	50/104 (48.1%)	84/115 (73.0%)	<0.01*
Infection index				
WBC, ×10^9^/L	10.4 (8.6-12.8)	9.9 (7.7-12)	11.1 (9.5-13.9)	<0.01*
Neutrophil, ×10^9^/L	8.5 (6.7-10.6)	8.1 (5.9-10)	9.6 (7.9-12.5)	<0.01*
Lymphocyte, ×10^9^/L	1.1 (0.8-1.5)	1.3 (0.9-1.7)	1 (0.7-1.4)	<0.01*
Outcomes				
mRS at 90 days	3 (1-6)	1 (0-2)	6 (4-6)	<0.01*
Any intracranial hemorrhage	39/219 (17.8%)	12/104 (11.5%)	27/115 (23.5%)	0.02*
Death within 90 days	58/219 (26.5%)	0	58/115 (50.4%)	<0.01*

Data are presented as n/N (%) or median (IQR). Data were missing for TOAST classification in three patients. ACA, anterior cerebral artery; ASPECTS, Alberta Stroke Program Early CT Score; BA, basilar artery; bpm, beats per minute; DPT, door-to-puncture time; EVT, endovascular thrombectomy; IQR, interquartile range; IV, intravenous; MCA, middle cerebral artery; mRS, modified Rankin Scale; NIHSS, National Institutes of Health Stroke Scale; OPT, onset-to-puncture time; ORT, onset-to-reperfusion time; TOAST, Trial of Org 10172 in Acute Stroke Treatment; VA, vertebral artery; WBC, white blood cell count. Bold values and asterisks indicate *P* < 0.05.

Compared with patients with favorable outcomes, those with unfavorable outcomes had more severe neurological deficits at baseline (median NIHSS score, 17 vs 13; *P* < 0.01) and a lower frequency of anterior circulation occlusion (67.0% vs 79.8%; *P* = 0.03). The overall distribution of artery occlusion sites also differed between the two groups (*P* = 0.04). During hospitalization, pneumonia occurred more frequently in patients with unfavorable outcomes than in those with favorable outcomes (73.0% vs 42.3%; *P* < 0.01), and antibiotic administration was also more common (73.0% vs 48.1%; *P* < 0.01). Patients with unfavorable outcomes had higher admission white blood cell counts (11.1 vs 9.9 × 10^9^/L; *P* < 0.01) and neutrophil counts (9.6 vs 8.1 × 10^9^/L; *P* < 0.01), together with lower lymphocyte counts (1.0 vs 1.3 × 10^9^/L; *P* < 0.01). Other baseline and procedural characteristics were comparable between the two groups.

Overall, 39 patients (17.8%) developed intracranial hemorrhage, which occurred more frequently among those with poor outcomes (23.5% vs 11.5%; *P* = 0.02). All 58 deaths (26.5%) occurred in the poor-outcome group (*P* < 0.01).

Results of logistic regression analyses are shown in Table 2. Pre-EVT temperature was not significantly associated with 90-day favorable functional outcome, any intracranial hemorrhage, or death. In univariate analysis, each 1 °C increase in post-EVT temperature was associated with reduced odds of achieving favorable functional outcome (OR, 0.40; 95% CI, 0.20-0.79; *P* = 0.01) and higher odds of mortality (OR, 2.02; 95% CI, 1.11-3.66; *P* = 0.02). After adjusting for potential confounders, these associations were attenuated (adjusted OR for favorable outcome, 0.47; 95% CI, 0.19-1.15; *P* = 0.10; adjusted OR for mortality, 1.22; 95% CI, 0.49-3.02; *P* = 0.67). Post-EVT temperature was not significantly associated with any intracranial hemorrhage in either model.

**Table 2:** Logistic regression ORs of outcome measures by body temperature measurements.

Outcome measure	Univariate analysis		Multivariate analysis	
	*P* value	OR (95% CI)	*P* value	OR (95% CI)
Pre-EVT temperature (per 1 °C)				
mRS 0-2 at 90 d	0.76	1.14 (0.51-2.54)	0.72	0.84 (0.32-2.18)
any intracranial hemorrhage	0.77	0.85 (0.29-2.52)	0.46	1.49 (0.52-4.31)
death within 90 d	0.71	0.84 (0.33-2.14)	0.65	1.30 (0.42-3.99)
Post-EVT temperature (per 1 °C)				
mRS 0-2 at 90 d	0.01*	0.40 (0.20-0.79)	0.10	0.47 (0.19-1.15)
any intracranial hemorrhage	0.65	0.84 (0.39-1.81)	0.32	0.52 (0.14-1.88)
death within 90 d	0.02*	2.02 (1.11-3.66)	0.67	1.22 (0.49-3.02)

Univariate and multivariate logistic regression analyses were performed to assess the association of body temperature and clinical outcomes. Body temperature was included in the model as a continuous variable, and its odds ratio (OR) represents the change in the odds of the outcome per 1 °C increase. The multivariate analysis was adjusted for the following potential confounding variables: age, sex, smoking, hypertension, diabetes mellitus, baseline NIHSS, ASPECTS, anterior circulation occlusion, TOAST, WBC. EVT indicates endovascular thrombectomy; mRS, modified Rankin Scale; OR, odds ratio. Bold values and asterisks indicate *P* < 0.05.

Ordinal shift analyses of 90-day mRS distributions are shown in Figures 2, 3. In the pre-EVT temperature analysis (Figure 2), outcome distributions were largely overlapping between patients with temperatures < 36.5 °C and ≥ 36.5 °C, without a discernible directional shift toward either better or worse outcomes. In contrast, post-EVT temperature stratification (Figure 3) revealed a rightward shift in the mRS distribution among patients with temperatures ≥ 36.6 °C, indicating a higher proportion of unfavorable functional outcomes compared with those whose post-procedural temperature was < 36.6 °C.

**Figure 2:**
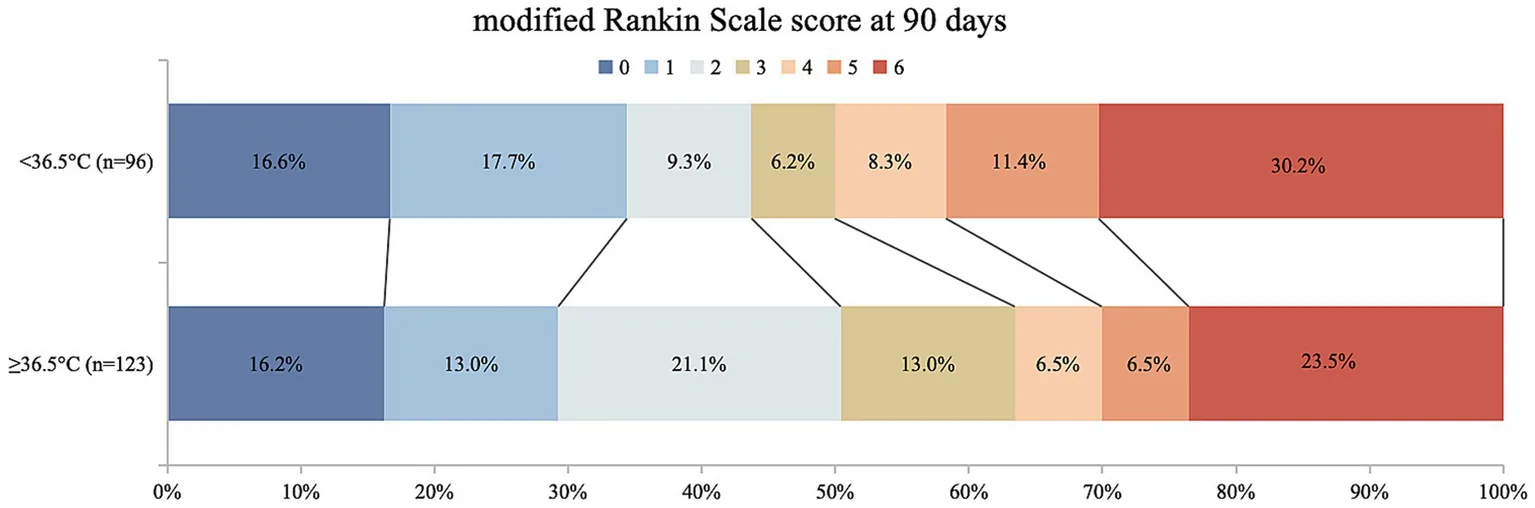
Ordinal shift analysis—comparison of 90-day mRS distribution between patients with pre-EVT temperature <36.5 °C and ≥36.5 °C.

**Figure 3:**
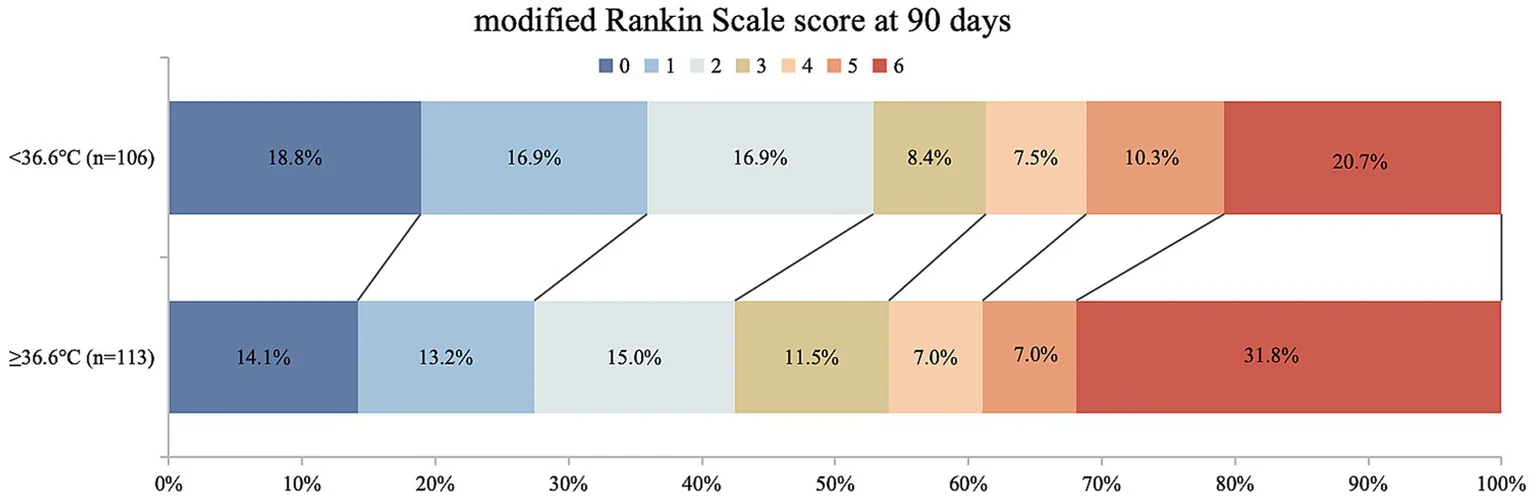
Ordinal shift analysis—comparison of 90-day mRS distribution between patients with post-EVT temperature <36.6 °C and ≥36.6 °C.

Exploratory subgroup analyses (Figure 4) were performed to assess whether the association between post-EVT temperature and favorable functional outcome was consistent across clinically relevant strata. Among patients with baseline NIHSS ≤ 15, a trend toward lower odds of functional independence was observed with each 1°C increase in post-EVT temperature after adjustment (adjusted OR, 0.29; 95% CI, 0.08-0.98; *P* = 0.047). This association was not observed in patients with NIHSS > 15 (adjusted OR, 0.82; 95% CI, 0.19-3.52; *P* = 0.79). The interaction between baseline NIHSS and post-EVT temperature was not statistically significant (*P* for interaction = 0.64).

**Figure 4:**
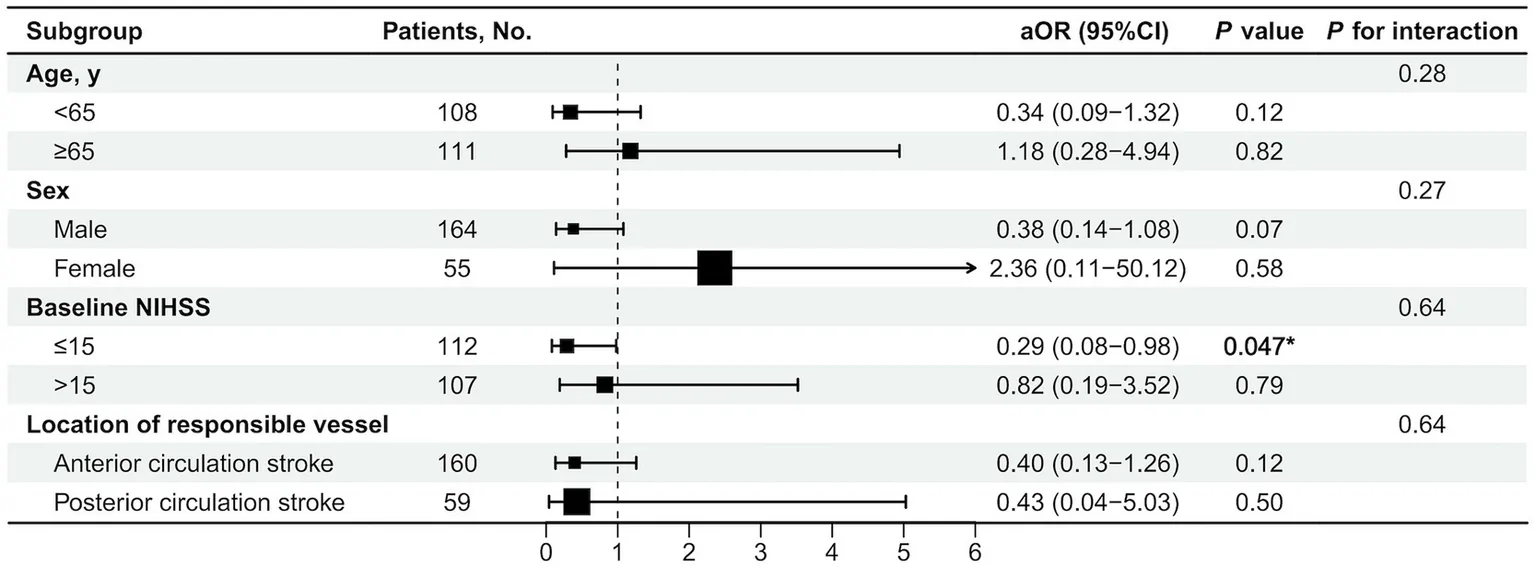
Forest plot illustrating the association of post-EVT body temperature with favorable outcome (defined as mRS score of 0-2) across subgroups. Adjusted variables: age, sex, smoking, hypertension, diabetes mellitus, baseline NIHSS, ASPECTS, anterior circulation occlusion, TOAST, WBC, excluding the corresponding stratification variable. mRS, modified Rankin Scale; NIHSS, National Institutes of Health Stroke Scale.

## 4 Discussion

This retrospective cohort study included patients with large-vessel-occlusion acute ischemic stroke (LVO-AIS) who underwent endovascular treatment (EVT) and achieved successful reperfusion. In unadjusted analyses, higher post-EVT body temperature was associated with worse functional outcome. However, this association was no longer statistically significant after adjustment for baseline stroke severity and inflammation-related clinical variables. These findings suggest that post-EVT temperature may not represent a stable prognostic factor independent of baseline disease burden.

Following vascular recanalization, secondary processes such as oxidative stress, endothelial dysfunction, blood-brain barrier disruption, inflammatory activation, and cerebral edema may influence neurological recovery (19–21), and higher temperature may amplify these responses (22–24). Thus, the association observed in unadjusted analyses is plausible. However, previous studies have shown that body temperature is strongly influenced by stroke severity and early complications. Boysen reported that early body temperature after acute stroke was determined by stroke severity and that admission temperature within 6 hours had no prognostic influence on 3-month outcome (25). Ruborg et al. (26) similarly found that temperature elevation within 48 hours after stroke was associated with admission NIHSS score and infection-related variables. These findings suggest that body temperature should not be interpreted as an isolated physiological marker, but rather as a clinical manifestation of neurological injury, systemic inflammation and early complications. Thus, it is understandable that the association between post-EVT temperature and outcome was attenuated after adjustment for NIHSS, ASPECTS, and inflammation-related covariates.

The timing of temperature measurement may also influence the results. Karaszewski et al. (27) reported that peak body temperature in ischemic stroke typically occurs 1.5-2 days after stroke onset and is more closely related to stroke severity, stroke subtype, and outcome than admission temperature. Geurts et al. (28) also reported that admission temperature was not associated with infarct volume or functional outcome, whereas higher peak temperatures during the first several days after stroke were associated with larger infarct volumes and poorer outcomes. Therefore, the absence of an independent association between post-EVT temperature and functional outcome may be partly attributable to limitations in temperature assessment. Although the 24-hour maximum post-EVT temperature was used, intermittent measurements may not fully capture temperature trajectories during the first 24-72 hours after stroke.

Several previous studies have evaluated body temperature in EVT-treated patients. Diprose et al. (29) reported that higher body temperatures during the peri-procedural period were associated with poorer outcomes after EVT. Chen et al. further focused on early temperature elevation within 24 hours after EVT and found that the peak body temperature (≥ 38 °C) was associated with symptomatic intracranial hemorrhage, death, and unfavorable functional outcome (30). Luo et al. demonstrated that the maximum body temperature within 24 hours after EVT was associated with early neurological deterioration (31). Building on these prior studies, the present study further focused on patients who had already achieved successful reperfusion. Our findings suggest that the independent prognostic value of post-EVT temperature may be limited in successfully reperfused patients. Its clinical interpretation should be integrated with stroke severity, imaging-defined tissue injury, inflammatory status, and other relevant clinical factors.

In the NIHSS ≤ 15 subgroup, a more pronounced association between post-EVT temperature and outcome was observed. However, the interaction between baseline NIHSS and post-EVT temperature did not reach statistical significance. Therefore, this subgroup finding should be interpreted with caution and considered hypothesis-generating. A consideration may help contextualize this subgroup finding. In patients with milder baseline neurological injury, the association between post-EVT body temperature and outcome may be easier to detect. By contrast, in patients with more severe baseline injury, the dominant influence of the initial neurological deficit may overshadow any additional prognostic contribution of post-EVT temperature. This hypothesis requires validation in larger cohorts with prespecified subgroup analyses.

Recently, a multicenter randomized pilot trial showed that intra-arterial selective hypothermia combined with EVT was feasible and safe, with a nonsignificant trend toward improved 90-day favorable outcome overall (mRS 0-2: 58.0% vs. 40.0%; *P* = 0.055) and a potential treatment signal in patients with baseline ASPECTS ≥ 8 (32). Although this finding aligns with observations from a prior trial, neither study provides definitive evidence that stroke severity or tissue injury status modifies the temperature–outcome relationship. Thus, this hypothesis remains exploratory and requires prospective validation in adequately powered studies with pre-specified subgroup analyses.

This study has several limitations. First, because this was a retrospective analysis, selection bias, information bias, and residual confounding cannot be fully excluded, even after multivariable adjustment. Second, peri-procedural temperature data from the other participating centers were unavailable, precluding comparison with the overall DETECT-China registry population. Therefore, the representativeness of the present cohort could not be determined, which may limit the generalizability of our findings. Third, the body temperature measurement method used in this study may not accurately reflect core body temperature. In addition, intermittent measurements at 4-hour intervals may have missed temperature fluctuations, and the 24-hour observation window may not have captured the overall post-stroke temperature peak, which often occurs beyond 24 hours. Fourth, admission WBC count was included as an inflammation-related covariate because it was measured before the 24-hour post-EVT temperature assessment. However, infection diagnoses were made during hospitalization, and some infections may have occurred after the 24-hour observation period. Therefore, infection status was not included in the multivariable analysis, and residual confounding related to infection cannot be excluded. Fifth, information on antipyretic use was incomplete, precluding reliable reporting or adjustment. Sixth, quantitative infarct volume data were unavailable, limiting adjustment for infarct size. Therefore, residual confounding related to infarct size cannot be excluded.

## 5 Conclusion

In this retrospective analysis of successfully reperfused patients with LVO-AIS, body temperature was not independently associated with functional outcome in the overall cohort. Although a potential association between higher post-EVT temperature and unfavorable outcome was observed in patients with baseline NIHSS ≤ 15, the interaction was not significant. This exploratory finding should be interpreted with caution and warrants prospective, pre-specified validation to clarify whether baseline stroke severity modifies the temperature-outcome relationship.

## Data Availability

The raw data supporting the conclusions of this article will be made available by the authors, without undue reservation.
